# The Effectiveness of a Mobile Health Clinic Delivering Mandatory and Elective Middle School Immunizations: A Descriptive Analysis

**DOI:** 10.7759/cureus.45452

**Published:** 2023-09-18

**Authors:** Natalie R LaGattuta, Taylor C Wilson, Jordan A Failla, Alexis M Stoner, Karen Fradua, Jaime Brown, Sheri C Byrd, Angela Wilson, Doralyn Jones, Lisa Carroll

**Affiliations:** 1 Preventive Medicine and Public Health, Edward Via College of Osteopathic Medicine, Spartanburg, USA; 2 Epidemiology and Public Health, South Carolina Department of Health and Environmental Control, Columbia, USA; 3 Pediatrics, Spartanburg Regional Healthcare System, Spartanburg, USA; 4 Pediatrics, Byrd Medical Associates, Spartanburg, USA; 5 Data Quality Analysis, Mayo Clinic, Spartanburg, USA; 6 Family Medicine, Edward Via College of Osteopathic Medicine, Spartanburg, USA

**Keywords:** preventive medicine, hpv, tdap, immunizations, mobile clinic

## Abstract

Introduction: Mobile health clinics serve a unique role in which they can offer affordable and adaptable care to the population they serve. The Edward Via College of Osteopathic Medicine (VCOM) mobile clinics began in 2020 as a partnership with the South Carolina Department of Health and Environmental Control (SCDHEC) to address the low vaccination rates that resulted from the COVID-19 pandemic.

Methods: This study is a descriptive analysis that examines the number of vaccinations of tetanus, diphtheria, and pertussis (Tdap) and human papillomavirus (HPV) at different locations of administration including pediatrician offices, the novel VCOM mobile vaccination clinic, and the Spartanburg Health Department. The variables of interest and the study endpoints focused on Tdap and HPV vaccinations among students aged 11-12 years old in Spartanburg County according to the type of healthcare delivery location.

Results: From April to May of 2021, the VCOM mobile clinic was able to administer 279 Tdap vaccines and 189 HPV vaccines to students at local middle schools, which surpasses the number of vaccines administered at other sites from August 2020 to May 2021 when compared individually for both Tdap and HPV vaccinations.

Conclusions: By assessing the total volume of vaccines administered by each group, the VCOM mobile clinic was established as an effective method of delivery and played a crucial role in the vaccination efforts of the Spartanburg community. Mobile medical units should be considered for similar efforts in providing care to resource-limited communities and those with limited access to care.

## Introduction

With the start of the COVID-19 pandemic in 2020, many primary care offices began limiting in-person visits and offered telemedicine visits to combat the spread of the virus [1]. Due to the closure of pediatric offices in response to COVID-19, there were significant decreases in the number of provider vaccine orders and doses administered for all age groups nationwide [2]. Many parents and children relied on inpatient visits with their primary care physicians to obtain mandatory immunizations for entry to school. In South Carolina (SC) specifically, many schools moved to online learning to prevent outbreaks within the classrooms; however, vaccination requirements remained in place. Therefore, the closure of pediatric offices and the move to online learning created a unique public health dilemma. As a result, from January to September 2020, there was a 26% decrease in measles, mumps, and rubella (MMR) vaccines; tetanus, diphtheria, and pertussis (Tdap) vaccines; and polio vaccines with a projected nine million doses missed by the end of 2020 [3]. By May 2020, weekly vaccine administration for children <24 months recovered to pre-pandemic levels; however, this was not the case for older children who were receiving two-thirds of the weekly vaccines during the same time frame prior to the pandemic in 2019 [4].

In addition to the impact of COVID-19 on immunization rates, South Carolina was already facing barriers to immunization delivery, such as an exponential rise in religious exemptions. For the 2014-2015 school year, there were a total of 5,826 religious exemptions throughout the state, with 0.79% of all students (N=778,588) having a religious exemption [5]. This number has continued to steadily increase with 11,154 exemptions, affecting 1.40% of students in 2018-2019 (N=796,384) [5]. Spartanburg County showed similar increases in the number of religious exemptions and percentage of students having a religious exemption from 2014-2015 to 2018-2019, with the number of religious exemptions increasing from 777 to 1,608 and the percentage of students with religious exemptions increasing from 1.59% (N=48,931) to 3.17% (N=50,683) [5]. From 2015 to 2019, Spartanburg County remained the county with the highest number of religious exemptions and the highest percentage of students with religious exemptions in SC [5]. A report published in 2019 claims that of the 11,000 religious exemptions from childhood vaccines in South Carolina, over one-third of cases are in Spartanburg County and Greenville County alone [5]. This represents an 18% (N=1,777) increase from the year prior.

In August 2020, to combat the already decreasing vaccination numbers further exacerbated by the pandemic, the Edward Via College of Osteopathic Medicine - Carolinas (VCOM-CC) formed a unique and impactful partnership with the SC Department of Health and Environmental Control (SCDHEC) utilizing VCOM’s mobile medical unit to provide vaccinations on school grounds, thus complying with COVID-19 policies that limited access to school buildings. The purpose of the VCOM-DHEC partnership was to reduce barriers and provide access to both mandatory and optional immunizations. This not only created a more convenient location for students to get their required vaccines but also created a safer setting for the students to receive the vaccines since most of the vaccinations were given outdoors with minimal human contact.

Mobile health clinics have proven to be helpful in the delivery of preventive health measures to vulnerable populations [6]. Mobile health clinics also serve a unique role in which they can offer affordable and adaptable care at a reduced cost to the population they serve [7]. As of 2017, there are more than 2,000 mobile health clinics nationwide, accounting for more than 6.5 million visits annually [8]. One advantage mobile clinics demonstrate is that they are effective at addressing barriers to care that many patients may face when seeking health services [7]. Such barriers include transportation, insurance, legal status, financial costs, linguistic/cultural barriers, and intimidation by healthcare settings, as well as many others [7]. By traveling directly to vulnerable communities, mobile health clinics can serve individuals who may have otherwise gone without care due to a lack of resources or due to the barriers listed above [9].

This study sought to determine if the VCOM mobile clinic was an effective modality in terms of the ability to deliver vaccinations in the Spartanburg community by assessing both mandatory and optional vaccination counts provided by the mobile unit as compared to other means of healthcare delivery such as the health department, outpatient hospital-affiliated pediatric office, and a private pediatric office. While this study does not allow for a direct comparison between modalities, obtaining this information can provide a better understanding and guide future discussions regarding the delivery of vaccinations to pediatric populations in South Carolina. 

## Materials and methods

Ethical design

This descriptive, retrospective study was approved as exempt, and consent was not required by the Edward Via College of Osteopathic Medicine Institutional Review Board (IRB) in Blacksburg, Virginia, USA. The IRB number assigned to this study is 1829548-2.

Study population

Using a convenience sampling approach, this study is based on the collection of vaccination counts for Tdap and first-dose human papillomavirus (HPV) vaccines for 11- to 12-year-olds from August 2020 to May 2021 at the VCOM mobile clinic sites, a local health department, an outpatient hospital-affiliated pediatric office, and a private pediatric office. The VCOM mobile clinic operated from August 2020 to May 2021 as part of a pilot study in partnership with DHEC. The mobile clinic provided various health services based on the needs of the population being served, including flu and COVID-19 vaccinations in the fall of 2020 and adolescent bundle vaccinations in the spring of 2021. While the adolescent bundle vaccinations were only administered by the mobile clinic from April to May of 2021, the timeline of August 2020-May 2021 was used for the private pediatric office, hospital-affiliated pediatric office, and local health department to mimic the normal patterns of vaccine administration more closely for these facilities, which provide year-round care to this population.

In addition to the required Tdap vaccination for entry into seventh grade, students are also eligible to receive the HPV vaccine, an optional vaccination for their age group. The HPV vaccine remains an optional vaccine for kids primarily due to HPV often not being seen as an immediate concern for the target age group of 11-12 years due to HPV being sexually transmitted as well as the cost associated with healthcare visits and regard for patient autonomy. In addition, the HPV vaccine has also been viewed as controversial despite evidence proving that the vaccine is both safe and effective at preventing HPV-related cancers later in life [10,11]. The meningococcal vaccine, another mandatory vaccine for this age group, was not included in this study due to variations in the age and timing of vaccination among the various healthcare modalities of this study.

For the VCOM mobile clinic, the data was collected by the SCDHEC Upstate Immunization Program through their registration and administrative process. The data collected included the total number of children served by the mobile clinic and the total number of vaccines administered. A total of 17 mobile health clinics were held from April 2021 to May 2021. The mobile clinics were coordinated with local schools in Spartanburg and surrounding communities and were held on school grounds after school hours. For the health department, the data collected was from the DHEC Statewide Immunization Online Network (SIMON). The data also includes the number of patients receiving a Tdap vaccine who also chose to receive the first dose of the HPV vaccine. For the outpatient hospital-based pediatric clinic, the data was collected from the electronic medical records by identifying well-child checks for patients ages 11-12 from August 1, 2020, to May 31, 2021, using Slicer Dicer. The data includes total counts of Tdap and HPV vaccines provided at that visit. For the private practice, the data was pulled from the electronic medical records utilizing the dates of the study, the age range, and ICD codes 90460 and 90461, which are codes specifically for vaccine administration. The collected data from each entity included total numbers for both Tdap and HPV vaccine recipients within the given age group of 11-12 years and the time frame of August 2020-May 2021 (except for the VCOM mobile clinic, which served from April 2021 to May 2021). No demographics were collected for this study, as demographics are not collected as part of the vaccine registry for DHEC.

Analysis

Descriptive analyses were performed assessing the total number of Tdap doses and the total number of HPV doses for each site. Additionally, the proportion of participants who received the Tdap vaccine and then subsequently received a first or second dose of the optional HPV vaccine was calculated for each site. Comparative analysis was not used for this study due to the inability to calculate rates because of variations in project timeline among groups. All collected data was de-identified, and multiple types of pediatric care were assessed in an attempt to eliminate bias.

## Results

From April 2021 to May 2021, the VCOM mobile clinic served over 301 children aged 11-12 years old. The mobile clinic administered 279 Tdap vaccines and 189 first-dose HPV vaccines (Table 1). Of those receiving a Tdap vaccine, 189 (67.7%) also chose to receive their first dose of the HPV vaccine during their visit.

**Table 1:** Total Number of Vaccines Administered by Method of Vaccine Delivery *Note: This value includes patients who received the first, second, or both doses of the HPV vaccine, whereas the other two groups are only the first dose. VCOM: Edward Via College of Osteopathic Medicine: DHEC: Department of Health and Environmental Control, Tdap: tetanus, diphtheria, and pertussis, HPV: human papillomavirus

	VCOM Mobile Clinic	Health Department (DHEC)	Private Pediatric Office	Outpatient Hospital-Affiliated Pediatric Office
Clinic Timeline	April 2021-May 2021	August 2020-May 2021	August 2020-May 2021	August 2020-May 2021
Total Tdap Doses	279	79	64	177
Total HPV Doses	189	53	67*	81*
Percentage of 11- to 12-year-old patients receiving Tdap who then received an HPV dose	67.74% (N=189)	67.09% (N=53)	100% (N=67)	45.76% (N=81)

The health department administered 79 Tdap vaccines and 53 first-dose HPV vaccines to children aged 11-12 years old from August 2020 to May 2021. Of those who received the Tdap vaccine, 53 (67%) also chose to receive the first dose of the HPV vaccine (Table 1).

The private pediatric office served 81 patients aged 11 years old from August 2020 to May 2021. A total of 64 Tdap vaccines, 42 first-dose HPV vaccines, and 25 second-dose HPV vaccines were administered to this group (Table 1). Of those receiving Tdap, only 59 were eligible for their first HPV dose, and 40 patients did receive their first dose of the HPV vaccine. Five additional patients who received the Tdap vaccine were eligible for their first HPV dose; however, they had private insurance and received a prescription for the HPV vaccine to be administered at a local pharmacy. Additionally, 13 patients who received their second dose of the HPV vaccine also received their first dose within the same time frame of data collection. Both HPV vaccination doses are included in the total and their respective count. A total of 67 HPV vaccines were administered during this time frame, and 100% of patients who received a Tdap vaccine also received either the first or second dose (or both) of the HPV vaccine.

The outpatient hospital-affiliated pediatric office had a total of 451 patient visits for patients aged 11-12 years old from August 2020 to May 2021 (Table 1). During this time frame, 177 patients received the Tdap vaccine. Of the 177 patients who received the Tdap vaccine, 81 received a dose of the HPV vaccine during their visit, and 96 patients did not receive any HPV vaccine; therefore, 45.8% of patients who received the Tdap vaccine also received a dose of HPV during their visit. However, of the 96 patients receiving Tdap who did not receive the HPV vaccine, 71 had already completed their HPV vaccination series. Of the other 25 patients receiving Tdap but not the HPV vaccine, three had received one dose of the HPV vaccine prior to their visit. Therefore, only 22 of the 177 patients who received Tdap had received no doses of the HPV vaccine.

## Discussion

Within the constraints of the study, it is evident that a mobile health unit is successful in administering both mandatory and optional vaccines for middle-school-aged children in SC. Even within the significantly shortened time frame, the VCOM mobile clinics received the highest number of total vaccine counts for both Tdap and HPV individually and combined for the number of vaccines administered compared to all other groups within this study. The percentage of students who received their first dose of HPV vaccine from the mobile clinic is also comparable to other groups. This data exemplifies the success and performance of the novel VCOM mobile clinic in supplying vaccines to students in the Spartanburg area.

The proportion of students who opted to receive a dose of the HPV vaccine after receiving the Tdap vaccine varied between each group. The outpatient hospital-based pediatric clinic had the lowest proportion of patients receiving HPV after their Tdap booster at 45.76% (N=81). However, 74% (71 of 96) of the patients who did not receive an HPV vaccine during our study time frame had already received the entire HPV vaccine series because patients at this practice begin HPV vaccination at age nine. Based on the understanding that the outpatient hospital-based clinic’s true HPV vaccination numbers are higher than the data from this study and that 100% of patients received both Tdap and HPV at the private pediatric office, it is possible that establishing a long-term relationship with a pediatrician is beneficial to vaccination efforts [12]. It is possible that the health department and the VCOM mobile clinic obtained similar proportions due to their purpose of serving populations that may not have access to routine care under a pediatrician; therefore, more patients were potentially eligible to receive these vaccinations during the timeline of the study compared to the pediatric offices. It can also be expected that the VCOM mobile clinic had a lower proportion of students receiving the HPV vaccine following their Tdap vaccine due to the primary purpose of the clinic being to provide mandatory vaccinations to the students.

Another implication of this study is the success of VCOM mobile clinics in terms of providing previously unoffered vaccines to students at their schools before the deadline. Prior to the COVID-19 pandemic, DHEC had school-located vaccination clinics with the purpose of administering the annual flu vaccine. The VCOM mobile clinic during COVID-19 was different in that it offered mandatory vaccines such as Tdap as well as optional vaccines such as the HPV series. This is significant because if a student was not up to date on their mandatory vaccines, then they were very likely also not up to date with optional vaccines offered to their age group. Although it is impossible to know why these students were previously not up to date on required vaccines so close to the deadline, it is likely that some of this could be attributed to a lack of access or other barriers to care [13]. Primary care physicians are usually seen on a yearly basis, and if a student was getting close to a deadline to receive their vaccine, it is likely that their doctor would have informed the student and guardian that they would need the required vaccine for school. This suggests that many of the students unvaccinated prior to the deadline either did not have a primary care provider or did not see them on a yearly basis [13].

Limitations

The mandatory Tdap vaccine is not recommended until 11 years old, which matched the age within our study. However, both pediatric clinics and the health department offer the optional HPV first dose at nine and the second dose following at age 10. Therefore, there were students in this study who had completed both doses and were not included in the vaccination counts as they had already received their HPV doses prior to their Tdap dose. This could lead to our data showing lower counts for HPV, when in fact it might be due to students having already had the dose prior to our study dates. Furthermore, for the health department and VCOM mobile clinic, we could expect the HPV vaccination levels to be higher if data including not only the first dose but also the second dose of the HPV series were available.

The COVID-19 pandemic was ongoing during our study time frame; however, for the outpatient hospital-based clinic and the health department, no restrictions were placed on office hours or the number of staff members. For the private pediatric office, COVID-19 restrictions from the end of March 2020 to April 2020 included seeing well visits in person and handling sick visits via phone or telehealth. The private practice was noted to have fewer well visits in the first two to three months. However, many calls were made to patients to bring them in for well visits during the beginning of the pandemic. Self-selection bias to attend the mobile unit should also be considered when analyzing the results. Those who chose to attend the mobile clinic may be unique compared to those who receive immunizations at a typical well-child visit. However, as this was a service in partnership with the schools, we feel that this was a representative sample of either those most in need of access to care, those who have other limitations in seeking immunizations, or families taking advantage of the convenience of the service at the school.

## Conclusions

Compared to all the other sites examined in the study, the mobile clinic was able to administer more Tdap and HPV vaccines from April 2021 to May 2021 than all other sites from August 2020 to May 2021. The novel VCOM mobile clinic proved itself an asset to the vaccination efforts of the Spartanburg community with an added benefit of convenience and potential reduction of barriers to care present within other healthcare delivery methods. This study should serve as a reference for future studies researching the benefits and efficacy of mobile clinics in rural areas. This study may also continue to guide the vaccination policy guidelines within Spartanburg County and possibly beyond. Although these clinics by no means replace the importance of a primary care physician for the students, future studies will likely demonstrate the massive potential mobile clinics have in terms of improving access in ways other than just vaccines, such as physicals and other preventative health measures. Future work is necessary post-pandemic to determine the driving factor(s) to the success of the VCOM mobile clinics, such as closure of medical offices, convenience, or ease of access, decreased barriers to care, or phobia of contracting COVID-19 at medical offices. Future studies should be performed to better understand how the mobile clinics address other social determinants of health such as access to and affordability of healthcare to improve vaccine efforts within the Spartanburg community.
